# Does the use of Doppler ultrasound reduce fetal mortality? A population study of all deliveries in Norway 1990–2014

**DOI:** 10.1093/ije/dyab098

**Published:** 2021-06-21

**Authors:** Jostein Grytten, Irene Skau, Anne Eskild

**Affiliations:** 1 Department of Community Dentistry, University of Oslo, Norway; 2 Department of Obstetrics and Gynecology, Institute of Clinical Medicine, Akershus University Hospital, Lørenskog, Norway

**Keywords:** Doppler ultrasound, fetal death, difference-in-difference estimates, obstetric care

## Abstract

**Background:**

The aim of the present study was to examine the effect that the introduction of Doppler ultrasound in obstetric care has had on fetal death in Norway. One mechanism by which Doppler ultrasound may reduce fetal death may be through the increased use of Caesarean delivery. Therefore, we also examined the effect that the use of Doppler ultrasound has had on the use of Caesarean delivery.

**Methods:**

The Medical Birth Registry of Norway provided detailed medical information for ∼1.2 million deliveries from 1990 to 2014. Information about the year of introduction of Doppler ultrasound was collected directly from the maternity units, using a questionnaire. The data were analysed using a hospital fixed-effects regression model with fetal death as the outcome measure. The key independent variable was the introduction of Doppler ultrasound at each maternity ward. Hospital-specific trends and risk factors of the mother for fetal death were included as covariates.

**Results:**

For pre-term deliveries, the introduction of Doppler ultrasound contributed to a reduction in fetal death of ∼30% and to an increase in planned Caesarean section of ∼15%. There were no effects for emergency Caesarean sections or inductions pre-term. The introduction of Doppler ultrasound had no effect on fetal death or Caesarean section for term deliveries.

**Conclusions:**

The introduction of Doppler ultrasound during the 1990s and 2000s made a significant contribution to the decline in the number of pre-term fetal deaths in Norway. Increased use of Caesarean section may have contributed to this reduction.

Key Messages

- During recent decades, there has been a significant decline in fetal death, particularly for pre-term deliveries.
- About 30% of the decline in pre-term fetal death can be explained by the introduction of Doppler ultrasound in antenatal care.
- The introduction of Doppler ultrasound contributed to an increase in planned Caesarean sections pre-term.
- The introduction of Doppler ultrasound has improved the identification of pregnancies with a high risk of fetal death and thereby enabled preventive interventions, such as Caesarean section.

## Introduction

The focus of the present study was to examine the effect that the introduction of Doppler ultrasound in obstetric care has had on fetal death in Norway. The causes of fetal death are not always known, but it is assumed that fetal death, at least to some extent, can be prevented by correct diagnosis and timely intervention of at-risk pregnancies.^1–3^ One commonly used fetal diagnostic tool is Doppler ultrasound.

Doppler ultrasound is used in high-risk pregnancies to examine feto–placental blood circulation.^4^ Changes in the pattern of blood flow in the umbilical artery may indicate abnormalities in the placenta. Intervention, e.g. Caesarean section, may then be needed. In Norway, high-risk pregnancies are identified at regular examinations at the hospital and/or at the primary antenatal-care clinics. Women at high risk are referred to the maternity ward at the hospital for further examination. Doppler ultrasound is then used as an additional measure performed during an ultrasound for fetal biometry. For further details about the Norwegian maternal services, see Supplementary Material Section 1, available as Supplementary data at *IJE* online.

There is no consensus on whether the use of Doppler ultrasound lowers the probability of the fetus being stillborn (for a review, see ^5–7^). For low-risk pregnancies, the conclusion from one of the most recent Cochrane reviews is that ‘Doppler ultrasound may have improved rates of stillbirth’.^5^ For high-risk pregnancies, the evidence is mixed.^6^^,^^7^ Imdad *et al.* (2011) summarized the findings from 16 randomized–controlled trials.^6^ They found that use of Doppler ultrasound led to a reduction in fetal deaths of 35% (*p* = 0.07). This result is not supported by Alfirevic *et al.* (2017), who concluded that there was no clear difference in the number of fetal deaths between pregnant women who had Doppler-ultrasound examination compared with those who had not.^7^ However, they point out that this result should be interpreted with caution, as the quality of the 19 trials included in the review was moderate to low. One weakness in the studies of high-risk populations was that the number of participants was too low to detect differences in outcome. Large sample sizes are needed because the prevalence of the outcome variable (fetal death) is low. Thus, the failure to identify an effect of Doppler ultrasound on stillbirth may be because of a lack of statistical power. Obstetric practice may also play a role. For example, it may be difficult to identify when use of Doppler ultrasound is indicated, as there is no consensus about how to define a high-risk pregnancy.

We examined our research question using a large and unique set of data that contains information about all fetal deaths and detailed medical information about all deliveries in Norway during the period 1990–2014. This was a period during which there had been a significant decline in fetal death in most Western countries, Norway included.^8–10^ During the same period, there was a rapid increase in the use of Doppler ultrasound. To what extent has the use of this diagnostic technology contributed to the decline in the fetal death rate? This decline has been particularly large for pre-term deliveries.^10^ Therefore, if Doppler ultrasound has an effect, we expect the effect to be particularly strong for pre-term deliveries. One mechanism by which Doppler ultrasound may reduce fetal death may be through increased use of Caesarean delivery. Therefore, we also examined the effect that the use of Doppler ultrasound had on the use of Caesarean delivery.

## Methods

### The source of the data

The analyses were carried out on data from the Medical Birth Registry of Norway (MBRN) for the period 1990–2014 (www.fhi.no). All deliveries are by law reported to the MBRN.^11^ Data from the MBRN were merged with two data registers. The first register contains information about the immigrant background of all first-generation immigrants.^12^ The second register, the Norwegian Standard Classification of Education, contains information about the highest education of all Norwegians.^13^

Information about the use of Doppler ultrasound was collected using a questionnaire that was sent to the head of every maternity unit in all the hospitals in the country. We asked them to provide the following information: ‘Enter as accurately as possible the year your maternity unit introduced the use of Doppler ultrasound.’ The response rate was high: 44 of 45 senior consultants replied. We restricted our analyses to the period 1990–2014, as Doppler ultrasound was first introduced in the early 1990s. In five maternity units, Doppler ultrasound had not been introduced by the end of 2014.

In the 45 maternity units that existed during the period 1990–2014, there were 1 317 155 deliveries (fetal deaths and live-born infants). In our analyses, we excluded all deliveries in the five maternity units in which Doppler ultrasound had not been introduced by the end of 2014 (*n* = 17 442) and multiple deliveries (*n* = 44 026). Then, our population encompassed 1 255 687 deliveries.

### The model specification

Our data structure consisted of a hospital-year panel. The control group comprised women who gave birth in a maternity unit before Doppler ultrasound was introduced. The treatment group comprised women who gave birth in the same maternity units after Doppler ultrasound was introduced. We defined a dummy variable (Doppler_Ultrasound), which had the value 0 for each year before Doppler ultrasound was introduced (= control group) and 1 for the year in which it was introduced and subsequent years (= treatment group). Our core regression model was then defined as:

$$Y_{\mathrm{ijt}}=\alpha +\beta_{1}D\mathrm{oppler}\_ultrasound_{jt}+\sum_{c}\gamma_{c}\cdot \mathrm{Covari}{\mathrm{ate}}_{\mathrm{ijt}}^{c}+\sum_{j}\delta_{j}\cdot \mathrm{Hospita}l_{j}^{}+\phi \cdot t+\;\sum_{j}\eta_{j}\cdot \mathrm{Hospita}l_{j}^{}\cdot t+u_{\mathrm{ijt}}\;\;\;\;(1)$$

where *Y_ijt_* is a binary variable indicating a stillborn baby *i* delivered in hospital *j* in year *t* (from 1990 to 2014). A stillbirth is defined as a baby born with no signs of life at or after 28 completed weeks of gestation.^14^^,^^15^ Separate regression analyses were run for the following lengths of gestation: from 28 completed weeks to <37 completed weeks (pre-term), from 37 completed weeks to <42 completed weeks (term) and ≥42 completed weeks (post-term).^16^^,^^17^ In order to take account of potentially confounding effects, Equation (1) includes several controls (for description, see Supplementary Material Section 2, available as Supplementary data at *IJE* online).

In all our analyses, we clustered the standard errors at the hospital level to account for positive serial correlation and within-hospital correlation.^18^ In Equation (1), the Doppler coefficient gives the change in the probability of fetal death when Doppler ultrasound was introduced at the maternity unit, holding the other independent variables constant.^19^

### Supplementary analyses

In order to test the key assumptions of the analyses, we carried out three supplementary analyses.

#### Pre-trends

In our analyses, evidence for an effect of Doppler ultrasound on fetal death comes from a deviation from the linear trend captured by the parameter ϕ in Equation (1).^20^ The sharper the deviation from the trend, the more likely we are to discover an effect of Doppler ultrasound, if there is one. A sharp deviation may be identified if the coefficient for the pre-trend is close to 0. Conversely, a sharp deviation is more difficult to identify if the pre-trend is negative. In that case, the fetal death rate was already decreasing before Doppler ultrasound was introduced.

Using figures and regression analyses, we examined pre-trends in fetal death according to year of introduction of Doppler ultrasound and gestational age. The data were aggregated at the hospital level. The outcome variable was defined as the number of fetal deaths divided by the number of fetal deaths and live-born infants (= the proportion of fetal deaths per hospital). The explanatory variable was the number of years before Doppler ultrasound was introduced. Hospital fixed effects and control variables measured as proportions at the hospital level were included in the analyses.

#### A placebo test

One concern with our study is that our exposure variable may be correlated with other changes in obstetric care that have improved perinatal health during the same time period as when Doppler ultrasound was introduced. These changes may be unobservable or difficult to measure, such as improvements in the quality of care, or better fetal screening. With panel data, it is possible to examine whether significant coefficients for Doppler ultrasound could be biased due to unobservable variables. We did this by carrying out a placebo test.^21^^,^^22^ A placebo test provides counter evidence by applying our regression model to a context in which no effect of Doppler ultrasound should be detected. If an apparent effect had been detected, that would have indicated that our results were biased.

We carried out our placebo test by redefining Equation (1) to capture pre- and post-intervention effects. We defined the following independent variables.

The contemporaneous effect was defined as 1 in the year when Doppler ultrasound was introduced and 0 in all other years. The first lead dummy variable was equal to 1 if Doppler ultrasound had been introduced 1 or 2 years before it actually was introduced and 0 otherwise. The second lead dummy variable was equal to 1 if Doppler ultrasound had been introduced 3 or 4 years before it actually was introduced and 0 otherwise. The first lagged dummy was equal to 1 in the 2 or 3 years after Doppler ultrasound was actually introduced and 0 otherwise The second lagged dummy was equal to 1 in the 4 or 5 years after Doppler ultrasound was actually introduced and 0 otherwise.

In this regression, we did not expect the lead variables to have any effect on fetal death. This is because the lead variables were defined to include years when Doppler ultrasound was not introduced. For the lag variables, we expected Doppler ultrasound to have an effect on fetal death.

#### The timing of the introduction of Doppler ultrasound

One assumption of our analyses is that the timing of the introduction of Doppler ultrasound was as good as random with respect to the outcome variable. This assumption would be violated if the maternity units introduced Doppler ultrasound as a response to poor perinatal health of the babies within their catchment area. For example, there could be a negative relationship between fetal death and the year of introduction of Doppler ultrasound. In that case, the estimate $\beta_{1}$ could be upward biased. We examined whether this was the case by estimating a regression model with the proportion of fetal deaths at the hospital level as the outcome variable. The key explanatory variable was the year Doppler ultrasound was introduced. Hospital fixed effects and control variables measured as proportions at the hospital level were included in the analyses.

### A possible mechanism

#### Does the use of Doppler ultrasound lead to increased use of Caesarean section?

This hypothesis was tested by estimating Equation (1) where *Y_ijt_* was redefined to be a binary variable indicating a baby *i* delivered by Caesarean section at hospital *j* in year *t* (from 1990 to 2014). In addition, we examined the effect of the introduction of Doppler ultrasound on planned Caesarean sections, emergency Caesarean sections and inductions. This was examined by carrying out separate analyses for each of these three interventions.

## Results

### Descriptive statistics

**Figure 1:**
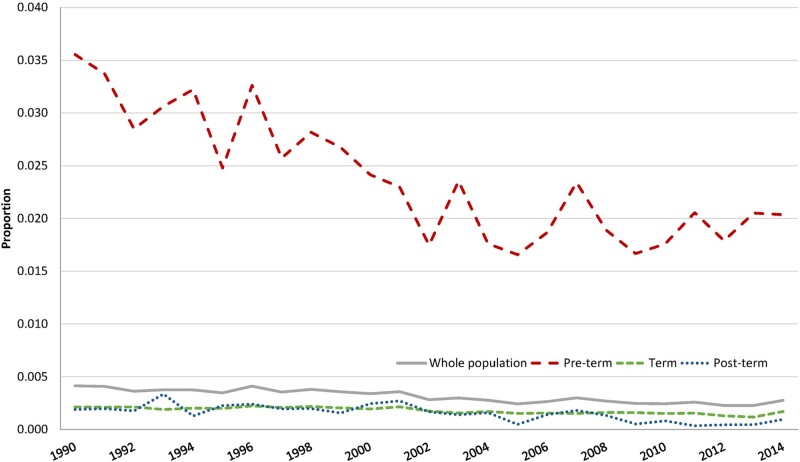
The proportion of fetal deaths according to year and gestational age. Aggregated data at the national level. 1990–2014.

During the period 1990–2014, in the population as a whole, there was a decline in the proportion of fetal deaths: from 0.41% to 0.28% (Figure 1). The greatest decline was for pre-term deliveries: from 3.5% to 2.0%. For term and post-term deliveries, there was only a minor decline in fetal deaths from 1990 to 2014.

**Figure 2:**
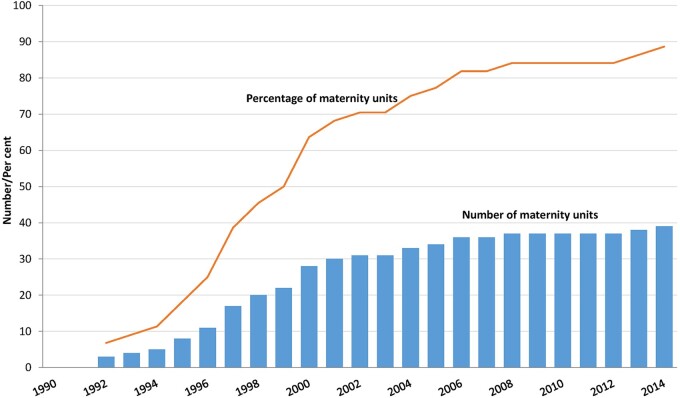
Number and percentage of maternity units, according to year of introduction of Doppler ultrasound. Cumulative figures. 1990–2014.

Doppler ultrasound was introduced for the first time in 1992 (Figure 2). By the second half of the 1990s, half of the maternity units had Doppler ultrasound. This increased to nearly 75% at the beginning of the 2000s. For pre-term deliveries, the graphs in Figures 1 and 2 show a clear trend of a negative relationship between the timing of the introduction of Doppler ultrasound and the decline in fetal deaths.

In Supplementary Material Section 3, available as Supplementary data at *IJE* online, we present descriptive statistics for the covariates, split by before and after the introduction of Doppler ultrasound. The split was made in two ways: first, 1 year before and 1 year after Doppler ultrasound was introduced; and, second, 1–2 years before and 1–2 years after Doppler ultrasound was introduced. The mean values of the covariates were similar before and after the introduction of Doppler ultrasound. For all the covariates, the 95% confidence interval (CI) overlapped.

### Effect of the use of Doppler ultrasound on fetal death

The regression coefficient was largest for pre-term deliveries (Table 1). For these deliveries, the coefficients were fairly similar independently of model specifications. In both models, with and without the interaction term *Hospital_j_* · *t*, the 95% CI for the coefficients overlapped (Table 1). This result is reassuring, as it indicates that a key assumption for identifying a causal effect has been fulfilled. In the model in which covariates, hospital-specific effects and hospital-specific linear trends were included, the use of Doppler ultrasound reduced the probability of fetal death by 0.0078 (95% CI, –0.01261 to –0.00303).

**Table 1:** The effects of the use of Doppler ultrasound on fetal deaths

			Gestational age period					
Variable	Whole population^a^		Pre-term^b^		Term^c^		Post-term^d^	
Doppler ultrasound	–0.00049	–0.00034	–0.00905	–0.00782	–0.00003	0.00005	0.00020	0.00026
P-value	0.025	0.106	0.000	0.002	0.858	0.713	0.610	0.552
95% CI	–0.00091 to –0.00006	–0.00076 to 0.00007	–0.01330 to –0.00481	–0.01261 to –0.00303	–0.00037 to 0.00031	–0.00026 to 0.00038	–0.00060 to 0.00101	–0.00063 to 0.00117
Covariates included	Yes	Yes	Yes	Yes	Yes	Yes	Yes	Yes
Linear trend (year of delivery)	Yes	Yes	Yes	Yes	Yes	Yes	Yes	Yes
Hospital fixed effects	Yes	Yes	Yes	Yes	Yes	Yes	Yes	Yes
Hospital fixed effects × linear trend	No	Yes	No	Yes	No	Yes	No	Yes
Number of fetal deaths	3671	3671	1693	1693	1802	1802	176	176
Total^e^	1 202 681	1 202 681	63 784	63 784	1 031 452	1 031 452	107 445	107 445

Single births. Regression coefficients clustered at the hospital level. 1990–2014.

a ≥28 completed weeks of gestation.

b From 28 completed weeks to <37 completed weeks of gestation.

c From 37 completed weeks to <42 completed weeks of gestation.

d ≥42 completed weeks of gestation.

e Includes number of live-born infants and number of fetal deaths.

In Supplementary Material Section 4, available as Supplementary data at *IJE* online, we report the results for deliveries at 28–33 weeks and at 34–36 weeks. For both lengths of gestation, the 95% CI for the regression coefficients overlapped.

In Supplementary Material Section 5, available as Supplementary data at *IJE* online, the results from the analyses in which maternal smoking was included as a covariate are reported. For all lengths of gestation, the 95% CI for the regression coefficients overlapped with the 95% CI for the coefficients from the analysis in which maternal smoking was not included (Table 1).

The regression coefficient for Doppler ultrasound for pre-term deliveries was 0.0078 (Table 1). During our study period of 1990–2014, the proportion of pre-term fetal deaths was 0.026. The reduction in fetal deaths, expressed in per cent, can be obtained by dividing 0.0078 by 0.026 and multiplying by 100%, which equals 30%.

For term and post-term deliveries, the regression coefficients were small, particularly for term deliveries. For all coefficients, the 95% CIs included the value 0. The regression coefficients for the covariates are reported in Supplementary Material Section 6, available as Supplementary data at *IJE* online. They had the same sign and were of similar size as previously reported in the literature.^1^^,^^2^^,^^23^

### Supplementary analyses

#### Pre-trends

**Figure 3:**
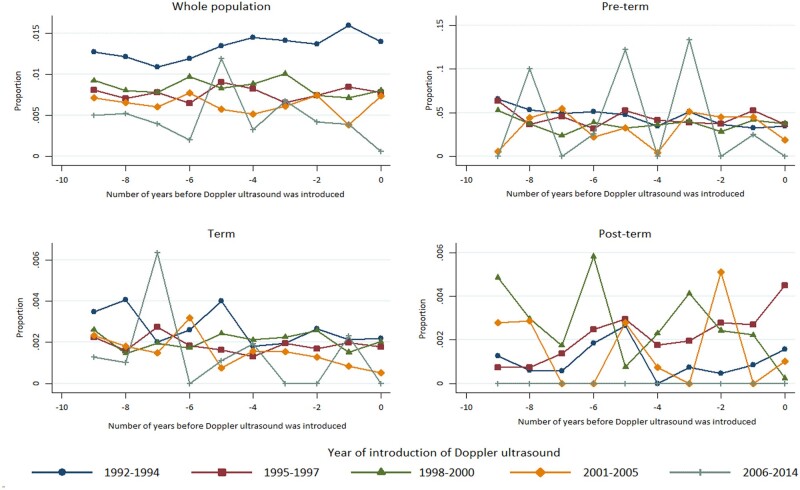
Trends in the proportion of fetal deaths before Doppler ultrasound was introduced. Single births, hospital-level data.

In Figure 3, we present the results of the analysis of pre-trends.

The vertical axis displays the proportion of fetal deaths at the hospital level. The horizontal axis displays the number of years before Doppler ultrasound was introduced. The hospitals were grouped according to the year in which Doppler ultrasound was introduced: 1992–1994 (early), 1995–1997, 1998–2000, 2001–2005, 2006–2014 (late). The key message from looking at these figures is that the lines are horizontal, i.e. the pre-trends are close to 0. This is supported by the results from the regression analyses (Supplementary Material Section 7, available as Supplementary data at *IJE* online). The coefficients were small and, for all coefficients, the 95% CIs included the value 0.

In our main analyses, we found an effect of Doppler ultrasound on pre-term deliveries (Table 1). We believe this result is reliable, as our supplementary analyses showed that there was a level trend in fetal death before the introduction of Doppler ultrasound.

#### Placebo test

**Figure 4:**
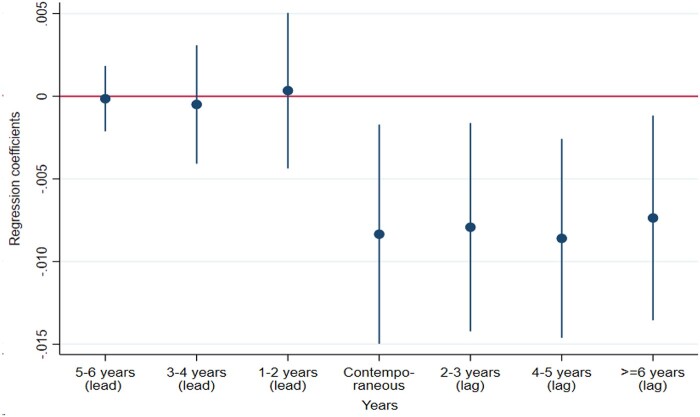
Lead and lag effects for the use of Doppler ultrasound on fetal deaths for pre-term deliveries. Single births. Regression coefficients with 95% confidence intervals. Covariates included. 1985–2014.

For pre-term deliveries, the estimates for the lead variables were small (Figure 4). For all coefficients, the 95% CIs included the value 0. This indicates that the results were not biased due to unobserved confounders. The coefficients for the lag variables were of a reasonable size, they had the expected sign (negative) and the 95% CIs did not include the value 0.

#### The timing of the introduction of Doppler ultrasound

The year that Doppler ultrasound was introduced had no effect on the response variables (Table 2). The regression coefficients were small and, for all coefficients, the 95% CIs included the value 0. This indicates that the results reported in Table 1 are not biased due to correlation between the timing of the introduction of Doppler ultrasound and the response variables.

**Table 2:** The effect of the year in which Doppler ultrasound was introduced on the proportion of fetal deaths

		Gestational age period		
Variable	Whole population^a^	Pre-term^b^	Term^c^	Post-term^d^
Year Doppler ultrasound was introduced	–0.00004	0.00036	–0.00002	–0.00006
*P*-value	0.145	0.688	0.262	0.159
95% CI	–0.00011 to 0.00002	–0.00143 to 0.00215	–0.00005 to 0.00001	–0.00013 to 0.00002
Number of observations (hospital-years)	364	358	364	356
Mean proportion of fetal deaths	0.00341	0.03924	0.00185	0.00189

Single births. Hospital-level data. Regression coefficients clustered by hospital. 1990–2014.

Covariates and hospital fixed effects were included in all the analyses.

Proportion of fetal deaths: number of fetal deaths divided by the number of fetal deaths and live-born infants.

a ≥28 completed weeks of gestation.

b From 28 completed weeks to <37 completed weeks of gestation.

c From 37 completed weeks to <42 completed weeks of gestation.

d ≥42 completed weeks of gestation.

#### Does the use of Doppler ultrasound lead to increased use of Caesarean section?

For pre-term deliveries, Doppler ultrasound had a positive effect on the probability of having a Caesarean section (Table 3). Depending on model specification, the use of Doppler ultrasound increased the probability of having a Caesarean section in the range 0.023 (95% CI, 0.0013 to 0.0446) to 0.026 (95% CI, –0.0024 to 0.0544). During the period 1990–2014, the proportion of Caesarean sections in pre-term deliveries was 0.36 (Table 3). Evaluated at this proportion, our results imply that the use of Doppler ultrasound has contributed to an increase in Caesarean sections of ∼7% for pre-term deliveries. For term and post-term deliveries, the regression coefficients were small and, for all coefficients, the 95% CIs included the value 0.

**Table 3:** The effects of the use of Doppler ultrasound on Caesarean section

			Gestational age period					
Variable	Whole population^a^		Pre-term^b^		Term^c^		Post-term^d^	
Doppler ultrasound	0.0078	0.0069	0.0260	0.0230	0.0044	0.0034	0.0037	0.0043
*P*-value	0.079	0.072	0.072	0.038	0.265	0.331	0.494	0.496
95% CI	–0.0009 to 0.0165	–0.0006 to 0.0143	–0.0024 to 0.0544	0.0013 to 0.0446	–0.0034 to 0.0122	–0.0035 to 0.0102	–0.0071 to 0.0146	–0.0083 to 0.0169
Year of delivery (*t*)	0.00094	0.00223	–0.00054	0.00101	0.00113	0.00241	0.00356	0.00575
*P*-value	0.007	0.000	0.605	0.058	0.001	0.000	0.000	0.000
95% CI	0.00027 to 0.00161	0.00188 to 0.00258	–0.00265 to 0.00156	–0.00003 to 0.00206	0.00050 to 0.00175	0.00208 to 0.00273	0.00267 to 0.00445	0.00498 to 0.00653
Covariates included^e^	Yes	Yes	Yes	Yes	Yes	Yes	Yes	Yes
Linear trend (year of birth)	Yes	Yes	Yes	Yes	Yes	Yes	Yes	Yes
Hospital fixed effects	Yes	Yes	Yes	Yes	Yes	Yes	Yes	Yes
Hospital fixed effects × linear trend	No	Yes	No	Yes	No	Yes	No	Yes
Number of Caesarean sections	168 653	168 653	23 136	23 136	130 375	130 375	15 142	15 142
Number of deliveries	1 202 681	1 202 681	63 784	63 784	1 031 452	1 031 452	107 445	107 445

Single births. Regression coefficients clustered at the hospital level. 1990–2014.

a ≥28 completed weeks of gestation.

b From 28 completed weeks to <37 completed weeks of gestation.

c From 37 completed weeks to <42 completed weeks of gestation.

d ≥42 completed weeks of gestation.

e The following covariates were included in the analyses: previous stillborn, previous Caesarean section, mother's age >35 years, parity, chronic disease, upper secondary education, university/college education, Non-European immigrant background, European immigrant background.

For pre-term deliveries, the effect of Doppler ultrasound was for planned Caesarean sections only (Supplementary Material Section 8, available as Supplementary data at *IJE* online). The use of Doppler ultrasound increased the probability of planned Caesarean sections by 0.011 (95% CI, –0.0002 to 0.0235). There were no effects for emergency Caesarean sections and inductions.

## Discussion

For pre-term deliveries, use of Doppler ultrasound reduced the number of fetal deaths. It is likely that the introduction of Doppler ultrasound has improved the identification of pre-term pregnancies at high risk of fetal death and thereby enabled preventive interventions, such as the use of Caesarean section. Increased use of Caesarean section may have contributed to this reduction. Most of the previous studies within this field are randomized controlled trials and clinical field studies.^5–7^ Our results are in line with the findings from these studies. For example, Imdad *et al.* (2011), in their review of 16 studies, found that the use of Doppler ultrasound led to a reduction in fetal death of 35% (*p* = 0.07).^6^ We found 30%, which is in the same order of magnitude.

There was no effect of Doppler ultrasound on fetal death for term and post-term deliveries (Table 1). This is as expected, as there was only a small decline in fetal deaths for these deliveries during our study period (Figure 1). The fetal death rates for term and post-term deliveries were low, at ∼1.7 per 1000 deliveries (Table 1).^24^ Another explanation for the lack of an effect of Doppler ultrasound on fetal death may be because Doppler ultrasound is not used as a standard diagnostic tool in term and post-term pregnancies. An exception is pregnancies for pregnant women who have pre-eclampsia. In Supplementary Material Section 9, available as Supplementary data at *IJE* online, we show the number of deliveries and the number of fetal deaths for this group of pregnant women. The number of fetal deaths is low, at only 54 deaths during the 25-year study period. This number is too low to identify any effect of Doppler ultrasound on fetal death in our regression analyses. To our knowledge, there are no other well-defined groups in which Doppler ultrasound is being used in term and post-term pregnancies. If such groups could be identified, the use of Doppler ultrasound could possibly be an effective way to cause a further reduction in the number of fetal deaths.

It was not possible with our data to distinguish between pregnant women who had been examined with Doppler ultrasound compared with those who had not. Such data are difficult to obtain. However, we managed to obtain such data from Akershus University Hospital (Ahus), which is one of the hospitals in Norway with the most deliveries. The data covered the period 2008–2014. On average, the proportion of pregnant women who had at least one examination with Doppler ultrasound was 0.37 throughout the period. There was little variation from year to year (Supplementary Material Section 10, available as Supplementary data at *IJE* online). This figure indicates that the use of Doppler ultrasound is an important diagnostic tool for the large hospitals. High-risk pregnant women are referred to the large hospitals for examination and delivery.^25^ Therefore, we would expect less use of Doppler ultrasound in small hospitals.

In conclusion, our study showed that the introduction of Doppler ultrasound during the 1990s and 2000s made a significant contribution to the decline in the number of fetal deaths in Norway. The decline was for pre-term deliveries only.

## Supplementary data

Supplementary data are available at *IJE* online.

## Ethics approval

The project was approved by the Norwegian Regional Committee for Medical and Health Research Ethics with registration number 2012/1433.

## Funding

This work was supported by the South-Eastern Norway Health Authority [research grant number 273903] (Biological responses to hypoxia: population studies of human pregnancy).

## Data availability

The data underlying this article will be shared on reasonable request to the corresponding author.

## Supplementary Material

dyab098_Supplementary_Data
