# Supplementary material for: Does the use of Doppler ultrasound reduce fetal mortality? A population study of all deliveries in Norway 1990–2014
Source: Int J Epidemiol. 2021 Jun 21;50(6):2038–47. doi: 10.1093/ije/dyab098 (PMC8743111; doi:10.1093/ije/dyab098)
Supplement: dyab098_Supplementary_Data [file dyab098_supplementary_data.pdf]

SUPPLEMENTARY MATERIAL

Section 1

The institutional setting for maternity services in Norway

In Norway, all health services, maternity care included, are financed through taxes. Government policy is that everyone is entitled to free health care at the point of delivery and there is equal access given equal need.[1] Nearly all deliveries take place in hospitals. Only 0.3% of all deliveries take place at home.[2] Hospitals are publicly owned and financed, with obstetricians who receive a fixed salary.

Antenatal health care is offered to all women free of charge. Almost 100% of pregnant women follow the programme from early pregnancy. From week nine of pregnancy until the expected date of delivery, mothers have at least eight antenatal clinical examinations with a midwife and/or a primary care physician.[3] During gestational weeks 17-18, all women have an ultrasound examination to determine fetal biometry and thereby gestational age, and to detect multiple fetuses and the localization of the placenta. The ultrasound examination is provided at the hospital in which the delivery is going to take place. Doppler ultrasound is not used at this examination.

References

1. Ministry of Health. *Behovsbasert finansiering av spesialisthelsetjenesten.* Oslo: Ministry of Health, 2002.
2. Blix E, Øian P, Kumle M. Utfall etter planlagte hjemmefødsler. *Tidsskr Nor Legeforen* 2008; 128: 2436-2439.
3. Norwegian Directorate of Health and Social Affairs. *A national clinical guideline for antenatal care. Short version - recommendations.* IS-1339/E. Oslo: Norwegian Directorate of Health and Social Affairs, 2005.

Section 2

Controls included in Equation (1)

$$Y_{ijt} = \alpha + \beta_1 Doppler\_ultrasound_{jt} + \sum_c \gamma_c \cdot Covariate_{ijt}^c + \sum_j \delta_j \cdot Hospital_j \; + \phi \cdot t + \sum_j \eta_j \cdot Hospital_j \; \cdot t + u_{ijt} \quad (1)$$

First, the equation includes fixed hospital effects (=*Hospital*$_j$). This was done in order to control for all time-invariant heterogeneity between hospitals, for example differences in the quality of obstetric care. At the hospital level we controlled for the number of pre-term deliveries. This takes into account that the prevalence of pre-term deliveries increased slightly during our study period.[1,2]

Second, the equation includes the year of delivery (= *t*), which controls for common and stabilizing trends that could affect the number of fetal deaths, for example better living standards and public health measures such as improved nutrition. To take into account that the time trend could have different effects for infants born in the different hospitals, hospital specific trends were also included using a set of interaction terms (= $Hospital_j \cdot t$). The assumption underlying the model without interaction terms is that the pre-trends in foetal death between hospitals are parallel.[3] This assumption is relaxed in the model with interaction terms, in which we allow the pre-trends to vary between hospitals. If the coefficients for Doppler ultrasound are different for the two models, this would indicate that the parallel trend assumption has not been fulfilled.

Third, Equation (1) includes covariates for several risk factors of the mother. These are variables that are well described in the literature, and that have been shown to be correlated with fetal death.[4-6] The following covariates of the mother were included: whether she had previously had a fetus that had died, whether she had previously had a Caesarean delivery, whether she was older (>35 years old) when she gave birth, parity, and whether she had a chronic disease. The mothers were classified as having a chronic disease if they had one or more of the following diseases: asthma, diabetes, epilepsy, heart disease, chronic hypertension, chronic kidney failure, and rheumatoid arthritis. Mothers with a high

level of education have fewer foetal deaths than mothers with a low level of education, and European immigrant mothers have fewer than non-European immigrant mothers. Therefore, these characteristics of the mothers were included as covariates in the analyses. Finally, we included the following pregnancy risk factors: gestational diabetes, pregnancy hypertension (gestational hypertension and preeclampsia), and placental abruption.

Fourth, Equation (1) includes a covariate for congenital abnormalities.[7,8] We constructed a binary variable that was given the value 1 for infants (stillborn and liveborn) who were diagnosed at birth as having a congenital abnormality, and 0 for those without. The fetuses were classified as having a congenital abnormality if they had one of the following: anencephalus, spina bifida, encephalocele, hydrocephalus, microtia, transposition of the great vessels, hypoplastic left heart, cleft palate without cleft lip, cleft lip with or without cleft palate, esophageal atresia, ano-rectal atresia, hypospadias, renal agenesis, limb reduction defects, diafragmatic hernia, ompalocele, gastroschisis, and Downs syndrome.

Maternal smoking is a risk factor for fetal death.[4-6] Information about maternal smoking was lacking for 45.8% of the pregnant women. Inclusion of maternal smoking as a covariate in our analyses would have led to a large reduction in the sample size. Therefore, we decided to carry out our main analysis without this variable included. However, we carried out a supplementary analysis in which we included maternal smoking as a covariate.

References

1. Tucker J, McGuire W. ABC of preterm birth. Epidemiology of preterm birth. *BMJ* 2004; 329: 675-678.

2. Goldenberg RL, Culhane JF, Iams JD, Romero R. Preterm birth 1. Epidemiology and causes of preterm birth. *Lancet* 2008; 371: 75-84.

3. Angrist JD, Pischke J-S. Differences-in-Differences. In: *Mastering metrics. The path from cause to effect*. Princeton, New Jersey: Princeton University Press, 2015: 178-208.

4. Gardosi J, Madurasinghe V, Williams M, Malik A, Francis A. Maternal and fetal risk factors for stillbirth: population based study. *BMJ* 2013; 346: f108. doi:10.1136/bmj.f108.

5. Flenady V, Koopmans L, Middleton P, et al. Major risk factors for stillbirth in high-income countries: a systematic review and meta-analysis. *Lancet* 2011; 377: 1331-1340.

6. The Stillbirth Collaborative Research Network Writing Group. *Association between stillbirth and risk factors known at pregnancy confirmation. JAMA 2011;* 306: 2469-2479.

7. Smith GCS, Fretts, RC. Stillbirth*.* *Lancet* 2007; 370: 1715-1725.

8. Getahun D, Ananth CV, Kinzler WL. Risk factors for antepartum and intrapartum stillbirth: a population-based study. *Am J Obstet Gynecol* 2007; 196: 499-507.

Section 3. Characteristics of the population before and after the introduction of Doppler ultrasound.
Hospital level.

Variable	Proportion/ mean 1 year before Doppler ultrasound was introduced	Proportion/ mean 1 year after Doppler ultrasound was introduced	Proportion/ mean 1 - 2 years before Doppler ultrasound was introduced	Proportion/ mean 1 - 2 years after Doppler ultrasound was introduced
Mother - demographic characteristics				
Mother's age > 35 years	0.0918	0.0963	0.0910	0.0991
95% CI	0.0845 to 0.0990	0.0883 to 0.1043	0.0861 to 0.0959	0.0937 to 0.1044
Upper secondary education	0.4257	0.4214	0.4261	0.4173
95% CI	0.4062 to 0.4366	0.4062 to 0.4366	0.4133 to 0.4389	0.4058 to 0.4289
University/college education	0.3164	0.3390	0.3131	0.3453
95% CI	0.2896 to 0.3433	0.3155 to 0.3625	0.2944 to 0.3319	0.3278 to 0.3627
Non-European immigrant background	0.0549	0.0563	0.0527	0.0593
95% CI	0.0411 to 0.0687	0.0447 to 0.0679	0.0435 to 0.0619	0.0507 to 0.0678
European immigrant background	0.0283	0.0286	0.0281	0.0302
95% CI	0.0210 to 0.0355	0.0232 to 0.0339	0.0236 to 0.0325	0.0265 to 0.0340
Mother - past pregnancy history				
Parity	0.9748	1.0093	0.9744	1.0054
95% CI	0.9300 to 1.0195	0.9596 to 1.0590	0.9410 to 1.0078	0.9720 to 1.0388
Previous Caesarean section	0.0780	0.0829	0.0769	0.0853
95% CI	0.0701 to 0.0858	0.0748 to 0.0910	0.0714 to 0.0825	0.0799 to 0.0908
Previous stillborn	0.0098	0.0092	0.0094	0.0091
95% CI	0.0080 to 0.0116	0.0078 to 0.0107	0.0083 to 0.0105	0.0080 to 0.0102
Mother - medical risk factors				
Chronic disease	0.0873	0.1075	0.0844	0.1066
95% CI	0.0719 to 0.1028	0.0867 to 0.1284	0.0748 to 0.0939	0.0936 to 0.1197
Gestational diabetes	0.0043	0.0081	0.0047	0.0073
95% CI	0.0030 to 0.0056	0.0045 to 0.0117	0.0033 to 0.0062	0.0053 to 0.0094
Pregnancy hypertension	0.0381	0.0444	0.0389	0.0466
95% CI	0.0325 to 0.0437	0.0387 to 0.0501	0.0352 to 0.0427	0.0426 to 0.506
Placental abruptions	0.0046	0.0046	0.0048	0.0041
95% CI	0.0037 to 0.0055	0.0036 to 0.0056	0.0040 to 0.0055	0.0035 to 0.0048
Fetus				
Congenital anomalies	0.0308	0.0296	0.0294	0.0298
95% CI	0.0250 to 0.0366	0.0239 to 0.0353	0.0257 to 0.0332	0.0258 to 0.0337
Hospital				
Number of pre-term deliveries	83.74	82.28	80.51	86.62
95% CI	56.33 to 111.15	56.33 to 108.24	62.00 to 99.01	67.69 to 105.56

Section 4. The effects of the use of Doppler ultrasound on fetal deaths according to length of gestation. Pre-term single births[1]. Regression coefficients clustered at the hospital level. 1990-2014

Variable	Gestational age period: From 28 to 33 completed weeks of gestation		Gestational age period: From 34 to 36 completed weeks of gestation	
Doppler ultrasound	-0.01508	-0.01673	-0.00498	-0.00331
P-value	0.061	0.064	0.009	0.095
95% CI	-0.03087 to 0.00070	-0.03450 to 0.00104	-0.00865 to -0.00132	-0.00724 to 0.00060
Covariates included	Yes	Yes	Yes	Yes
Linear trend (year of delivery)	Yes	Yes	Yes	Yes
Hospital fixed effects	Yes	Yes	Yes	Yes
Hospital fixed effects x linear trend	No	Yes	No	Yes
Number of fetal deaths	1 002	1 002	691	691
Total[2]	16 360	16 360	47 424	47 424

[1] From 28 completed weeks to less than 37 completed weeks of gestation

[2] Includes number of liveborn infants and number of fetal deaths

Section 5. The effects of the use of Doppler ultrasound on fetal deaths. Single births. Maternal smoking included as a covariate.
Regression coefficients clustered at the hospital level. 1990-2014

			Gestational age period					
Variable	Whole population[1]		Pre-term[2]		Term[3]		Post-term[4]	
Doppler ultrasound	-0.00014	-0.00057	-0.01252	-0.01674	0.00035	0.00016	0.00081	0.00081
P-value	0.765	0.244	0.082	0.040	0.345	0.693	0.329	0.407
95% CI	-0.00110 to 0.00082	-0.00155 to 0.00040	-0.02669 to 0.00164	-0.03265 to -0.00083	-0.00039 to 0.00110	-0.00068 to 0.00102	-0.00085 to 0.00249	-0.00115 to 0.00279
Covariates included	Yes	Yes	Yes	Yes	Yes	Yes	Yes	Yes
Linear trend (year of delivery)	Yes	Yes	Yes	Yes	Yes	Yes	Yes	Yes
Hospital fixed effects	Yes	Yes	Yes	Yes	Yes	Yes	Yes	Yes
Hospital fixed effects x linear trend	No	Yes	No	Yes	No	Yes	No	Yes
Number of fetal deaths	1 706	1 706	765	765	889	889	52	52
Total[5]	652 217	652 217	33 412	33 412	576 460	576 460	42 345	42 345

[1] 28 completed weeks or more of gestation
[2] From 28 completed weeks to less than 37 completed weeks of gestation
[3] From 37 completed weeks to less than 42 completed weeks of gestation
[4] 42 completed weeks or more of gestation
[5] Includes number of liveborn infants and number of fetal deaths

Section 6. The effects of the use of Doppler ultrasound on fetal deaths. Single births. Regression coefficients for the covariates.
Regression coefficients clustered at the hospital level. 1990-2014

Variable	Whole population[1]		Gestational age period: Pre-term[2]		Term[3]		Post-term[4]	
Doppler ultrasound	-0.00049	-0.00034	-0.00905	-0.00782	-0.00003	0.00005	0.00020	0.00026
P-value	0.025	0.106	0.000	0.002	0.858	0.713	0.610	0.552
95% CI	-0.00091 to -0.00006	-0.00076 to 0.00007	-0.01330 to -0.00481	-0.01261 to -0.00303	-0.00037 to 0.00031	-0.00026 to 0.00038	-0.00060 to 0.00101	- 0.00063 to 0.00117
Mother - demographic characteristics								
Mother's age > 35 years	0.00136	0.00137	0.00390	0.00388	0.00083	0.00083	0.00192	0.00190
P-value	0.000	0.000	0.047	0.049	0.000	0.000	0.003	0.004
95% CI	0.00102 to 0.00170	0.00103 to 0.00171	0.00005 to 0.00776	0.00001 to 0.00775	0.00056 to 0.00109	0.00057 to 0.00109	0.00067 to 0.00317	0.00065 to 0.00315
Upper secondary education	-0.00062	-0.00063	-0.00162	-0.00167	-0.00038	-0.00039	0.00062	0.00061
P-value	0.000	0.000	0.243	0.233	0.000	0.000	0.172	0.172
95% CI	-0.00089 to -0.00035	-0.00090 to -0.00036	-0.00440 to 0.00115	-0.00447 to 0.00112	-0.00058 to -0.00018	-0.00059 to -0.00019	-0.00028 to 0.00152	-0.00028 to 0.000152
University/college education	-0.00126	-0.00126	-0.00676	-0.00678	-0.00056	-0.00056	0.00040	0.00040
P-value	0.000	0.000	0.000	0.000	0.000	0.000	0.415	0.420
95% CI	-0.00149 to -0.00102	-0.00149 to -0.00103	-0.00958 to -0.00395	-0.00964 to -0.00393	-0.00079 to -0.00034	-0.00079 to -0.00034	-0.00058 to 0.00139	-0.00059 to 0.00139
Non-European immigrant background	0.00111	0.00111	0.00818	0.00821	0.00032	0.00033	0.00351	0.00352
P-value	0.000	0.000	0.004	0.004	0.114	0.111	0.001	0.001
95% CI	0.00059 to 0.00163	0.00058 to 0.00164	0.00271 to 0.01364	0.00271 to 0.01372	-0.00008 to 0.00074	-0.00008 to 0.00076	0.00145 to 0.00558	0.00143 to 0.00560
European immigrant background	0.00008	0.00009	0.00256	0.00277	-0.00009	-0.00009	0.00070	0.00072
P-value	0.775	0.736	0.273	0.254	0.648	0.673	0.345	0.330
95% CI	-0.00048 to 0.00064	-0.00047 to 0.00066	-0.00210 to 0.00722	-0.00207 to 0.00761	-0.00053 to 0.00033	-0.00053 to 0.00034	-0.00078 to 0.00220	-0.00076 to 0.00221
Mother - past pregnancy history								
Parity	-0.00007	-0.00006	0.00100	0.00102	-0.00007	-0.00006	-0.00001	-0.00001
P-value	0.297	0.337	0.086	0.077	0.128	0.161	0.955	0.952
95% CI	-0.00021 to 0.00006	-0.00020 to 0.00007	-0.00014 to 0.00216	-0.00011 to 0.00216	-0.00016 to 0.00002	-0.00016 to 0.00002	-0.00051 to 0.00048	-0.00051 to 0.00048
Previous Caesarean section	0.00043	0.00043	-0.00068	-0.00063	0.00006	0.00006	0.00095	0.00095
P-value	0.011	0.012	0.696	0.714	0.614	0.632	0.206	0.207
95% CI	0.00010 to 0.00077	0.00010 to 0.00076	-0.00419 to 0.00282	-0.00412 to 0.00285	-0.00019 to 0.00033	-0.00020 to 0.00033	-0.00054 to 0.00245	-0.00054 to 0.00245
Previous stillborn	0.00286	0.00281	0.00273	0.00284	0.00101	0.00097	-0.00019	-0.00023
P-value	0.009	0.010	0.586	0.569	0.107	0.122	0.931	0.916
95% CI	0.00077 to 0.00495	0.00072 to 0.00491	-0.00732 to 0.01278	-0.00719 to 0.01288	-0.00022 to 0.00226	-0.00027 to 0.00223	-0.00463 to 0.00425	-0.00468 to 0.00421
Mother - medical risk factors								
Chronic disease	0.00032	0.00033	-0.00409	-0.00408	0.00026	0.00027	0.00050	0.00051
P-value	0.034	0.028	0.033	0.034	0.042	0.034	0.334	0.328
95% CI	0.00002 to 0.00062	0.00003 to 0.00063	-0.00783 to -0.00034	-0.00785 to -0.00032	0.00001 to 0.00051	0.00002 to 0.00052	-0.00054 to 0.00155	-0.00053 to 0.00157
Gestational diabetes	0.00034	0.00031	-0.00744	-0.00762	0.00095	0.00092	-0.00213	-0.00221
P-value	0.475	0.513	0.040	0.037	0.040	0.046	0.000	0.000
95% CI	-0.00062 to 0.00130	-0.00065 to 0.00128	-0.01454 to -0.00035	-0.01475 to -0.00049	0.00004 to 0.00186	0.00001 to 0.00184	-0.00300 to -0.00125	-0.00310 to -0.00131

Variable	Whole population[1]		Gestational age period					
			Pre-term[2]		Term[3]		Post-term[4]	
Pregnancy hypertension	0.00085	0.00083	-0.01235	-0.01234	0.00001	0.0000004	0.00258	0.00260
P-value	0.002	0.003	0.000	0.000	0.949	0.999	0.047	0.045
95% CI	0.00032 to 0.00137	0.00031 to 0.00136	-0.01536 to -0.00933	-0.01537 to -0.00932	-0.00044 to 0.00047	-0.00046 to 0.00046	0.00003 to 0.00514	0.00006 to 0.00515
Placental abruptions	0.06301	0.06303	0.04982	0.04988	0.05736	0.05737	0.04661	0.04659
P-value	0.000	0.000	0.000	0.000	0.000	0.000	0.011	0.012
95% CI	0.05224 to 0.07378	0.05226 to 0.07380	0.03279 to 0.06684	0.03290 to 0.06687	0-04739 to 0.06732	0.04740 to 0.06733	0.01109 to 0.08212	0.01107 to 0.08211
Fetus								
Congenital anomalies	0.00283	0.00284	0.01436	0.01442	0.00025	0.00026	0.00059	0.00062
P-value	0.000	0.000	0.005	0.005	0.384	0.371	0.431	0.409
95% CI	0.00135 to 0.00432	0.00134 to 0.00434	0.00454 to 0.02418	0.00457 to 0.02427	-0.00033 to 0.00083	-0.00032 to 0.00085	-0.00091 to 0.00210	-0.00089 to 0.00215
Hospital								
Number of pre-term deliveries	0.000005	0.000008	-0.000002	0.000001	0.000003	0.000004	0.000006	0.000006
P-value	0.003	0.000	0.890	0.953	0.098	0.006	0.002	0.028
95% CI	0.000009 to 0.000008	0.000004 to 0.00001	-0.00003 to 0.00003	-0.00003 to 0.00003	-0.0000005 to 0.000006	0.000001 to 0.000008	0.000002 to 0.00001	0.0000007 to 0.000012
Linear trend (year of delivery)	Yes	Yes	Yes	Yes	Yes	Yes	Yes	Yes
Hospital fixed effects	Yes	Yes	Yes	Yes	Yes	Yes	Yes	Yes
Hospital fixed effects x linear trend	No	Yes	No	Yes	No	Yes	No	Yes
Number of fetal deaths	3 671	3 671	1 693	1 693	1 802	1 802	176	176
Total[5]	1 202 681	1 202 681	63 784	63 784	1 031 452	1 031 452	107 445	107 445

[1] 28 completed weeks or more of gestation

[2] From 28 completed weeks to less than 37 completed weeks of gestation

[3] From 37 completed weeks to less than 42 completed weeks of gestation

[4] 42 completed weeks or more of gestation

[5] Includes number of liveborn infants and number of fetal deaths

Notes. Reference categories: Mother's age ≤ 35 years. Education: compulsory school education.

Section 7. Test of pre-trends in fetal deaths according to year of introduction of Doppler ultrasound. Regression coefficients clustered at the hospital level.
Independent variable: number of years before the Doppler ultrasound was introduced. Single births. Hospital level data. Covariates included

Sample	Regression coefficient	P-value	95% CI
**Whole population**			
Year of introduction of Doppler ultrasound			
1992-1994	-0.00029	0.514	-0.00143 to 0.00084
1995-1997	-0.00034	0.050	-0.00070 to 0.0000007
1998-2000	-0.00005	0.934	-0.00150 to 0.00139
2001-2005	-0.00004	0.940	-0.00147 to 0.00138
2006-2014	-0.00037	0.114	-0.00088 to 0.00014
**Pre-term**			
Year of introduction of Doppler ultrasound			
1992-1994	-0.00728	0.052	-0.01466 to 0.00008
1995-1997	-0.00056	0.728	-0.00408 to 0.00294
1998-2000	0.00171	0.178	-0.00092 to 0.00435
2001-2005	0.00190	0.111	-0.00062 to 0.00443
2006-2014	0.00443	0.523	-0.01317 to 0.02203
**Term**			
Year of introduction of Doppler ultrasound			
1992-1994	0.00001	0.982	-0.00115 to 0.00117
1995-1997	-0.00011	0.446	-0.00042 to 0.00020
1998-2000	0.000007	0.989	-0.00110 to 0.00112
2001-2005	0.00006	0.807	-0.00062 to 0.00076
2006-2014	-0.00075	0.190	-0.00209 to 0.00057
**Post-term**			
Year of introduction of Doppler ultrasound			
1992-1994	0.00015	0.691	-0.00086 to 0.00118
1995-1997	0.00053	0.172	-0.00026 to 0.00133
1998-2000	-0.00028	0.521	-0.00123 to 0.00066
2001-2005	-0.00014	0.771	-0.00138 to 0.00109
2006-2014[1]	-	-	-

[1]Few observations

Section 8. The effects of the use of Doppler ultrasound on planned Caesarean sections, emergency Caesarean sections and inductions. Pre-term single births[1]. Regression coefficients clustered at the hospital level. 1990-2014

Variable	Planned Caesarean sections	Emergency Caesarean sections	Inductions
Doppler ultrasound	0.0116	0.0056	0.0026
P-value	0.055	0.581	0.824
95% CI	-0.0002 to 0.0235	-0.0147 to 0.0259	-0.0216 to 0.0270
Covariates included[2]	Yes	Yes	Yes
Linear trend (year of delivery)	Yes	Yes	Yes
Hospital fixed effects	Yes	Yes	Yes
Hospital fixed effects x linear trend	Yes	Yes	Yes
Number of Caesarean sections	5 215	17 769	8 888
Number of deliveries	63 784	63 784	63 784

[1] From 28 completed weeks to less than 37 completed weeks of gestation

[2] The following covariates were included in the analyses: previous stillborn, previous Caesarean section, mother's age > 35 years, parity, chronic disease, upper secondary education, university/college education, Non-European immigrant background, European immigrant background.

Section 9. The number of deliveries and the number of foetal deaths acording to year.
Pregnant women who have pre-eclampsia . Term and post-term pregnancies. Single births. 1990-2014

Year	Number of deliveries	Number of foetal deaths
1990	1 141	1
1991	1 179	3
1992	1 246	2
1993	1 119	1
1994	1 065	3
1995	1 054	1
1996	1 080	1
1997	987	1
1998	1 057	2
1999	1 633	5
2000	1 688	4
2001	1 618	7
2002	1 577	1
2003	1 416	5
2004	1 366	3
2005	1 454	3
2006	1 508	1
2007	1 404	0
2008	1 442	2
2009	1 325	2
2010	1 375	2
2011	1 241	1
2012	1 161	1
2013	1 089	2
2014	1 063	0
Total	32 288	54

Section 10. Proportion of pregant women who had an examination with Doppler ultrasound according to year (%). Akershus University Hospital. 2008 - 2014

Year	Number of pregnant women	Proportion who had an examination with Doppler ultrasound (%)
2008	4 284	39.3
2009	4 776	34.1
2010	4 716	37.0
2011	5 211	37.1
2012	5 340	36.0
2013	4 743	38.1
2014	5 177	38.2
Total	34 247	37.1
